# Long non-coding RNA XIST promotes cell growth and invasion through regulating miR-497/MACC1 axis in gastric cancer

**DOI:** 10.18632/oncotarget.13670

**Published:** 2016-11-28

**Authors:** Lei Ma, Yongjian Zhou, Xiaojun Luo, Hai Gao, Xubin Deng, Yingjie Jiang

**Affiliations:** ^1^ Cancer Hospital of Guangzhou Medical University, Guangzhou, China; ^2^ Department of Gastroenterology, the First Hospital of Guangzhou, Guangzhou, China; ^3^ Cancer Center, TCM-Integrated Hospital, Southern Medical University, Guangzhou, China; ^4^ Xiamen Hospital of Traditional Chinese Medicine, Xiamen, China; ^5^ Xiamen Hospital Affiliated to Fujian University of Traditional Chinese Medicine, Xiamen, China; ^6^ Department of Gastroenterology, Guangzhou Medical University, Guangzhou, China

**Keywords:** lncRNA XIST, miR-497, MACC1, gastric cancer

## Abstract

Abnormal expression of long non-coding RNA (lncRNAs) often contributes to unrestricted growth and invasion of cancer cells. LncRNA XIST expression is up-regulated in several cancers, however, its modulatory mechanism in gastric cancer (GC) has not been elucidated. In the present study, we found that XIST expression was significantly increased in GC tissues and cell lines. LncRNA XIST promoted cell cycle progression from the G1 phase to the S phase and protected cells from apoptosis, which contributed to GC cell growth. LncRNA XIST also contributed to GC cell invasion both *in vitro* and *in vivo*. We revealed that XIST functioned as competing endogenous RNA to repress miR-497, which controlled its down-stream target MACC1. We proposed that XIST was responsible for GC cell proliferation and invasion and XIST exerted its function through the miR-497/MACC1 axis. Our findings suggested that lncRNA XIST may be a candidate prognostic biomarker and a target for new therapies in GC patients.

## INTRODUCTION

Gastric cancer (GC) is the fourth most common cancer and the second leading cause of cancer-related deaths worldwide [1]. Many GC patients have already stepped into the late stage of the disease when they are diagnosed. In addition, metastasis after surgical resection and a high frequency of tumor recurrence contributes to a poor prognosis of GC patients [2]. Therefore, it is necessary to develop novel strategies for the diagnosis and treatment of GC.

Protein-coding genes account for about 2% of the human genome, whereas the majority of transcripts consist of non-coding RNAs (ncRNAs) [3]. Non-coding RNAs can be grouped into long non-coding RNAs (lncRNAs, > 200 nt) and small ncRNAs [4]. Abnormal expressions of lncRNAs often contribute to tumor initiation, metastasis and cell growth [5] and lncRNAs are dysregulated in many cancers, including GC [6]. LncRNA XIST is required for transcriptional silencing of one X-chromosome during female mammal development. It plays a significant role in the differentiation, proliferation, and genome maintenance of human cells [7] and abnormal expression often contributes to the development of human cancer [8]. For instance, lncRNA XIST expression is up-regulated in glioma tissues and promotes cell proliferation and invasion [9]. Furthermore, its overexpression is highly associated with occurrence, growth, invasion, and metastasis in ovarian cancer [10]. However, the role of lncRNA XIST in GC is still poorly understood.

MicroRNAs (miRNAs, 20-25 nt), a class of small ncRNAs, usually bind to the 3′-untranslated region (3′-UTR) of mRNAs which subsequently leads to mRNA degradation or translation repression [11]. It is well documented that alterations in miRNA expression play a critical role in cancer initiation and development [12, 13]. MiR-497 functions as a tumor suppressor and its expression is frequently down-regulated in cancer. Restoration of miR-497 expression abrogate tumorigenesis through inhibiting its down-stream targets, which are involved in the pathogenesis of cancer [14, 15]. In a previous study, we have demonstrated that miR-497 modulated GC cell proliferation and proposed that miR-497 was a tumor-suppressive microRNA in GC [16]. However, a more detailed role of miR-497 in the suppression of cell growth and invasion in GC has not been elucidated.

Recent literature has documented the interaction between lncRNA and miRNA [17]. This lncRNA-miRNA cross talk is involved in various human cancers. One of the potential mechanisms is that lncRNA is able to competitively inhibit miRNAs by acting as a molecular sponge [18]. For example, H19 lncRNA was shown to act as a molecular sponge for the let-7 family. On the other hand, let-7 could also decrease H19 expression [19]. In human gliomas, lncRNA CASC2 was able to decrease the expression of miR-21 while miR-21 can also suppress lncRNA CASC2 expression [20]. The cross talk between lncRNAs and microRNAs is also frequently found in GC. LncRNA HOTAIR is able to serve as a competing endogenous RNA (ceRNA) to sponge miR-331-3p, which accelerates the development and progression of GC [21]. The lncRNA H19/miR-675 pathway plays an oncogenic role in GC by regulating down-stream targets of miR-675 [6]. Recently, it was found that lncRNA MEG3 interacts with miR-148a in GC cells [22]. The cross-regulation between lncRNAs and microRNAs highlights their influences on the progression of GC and promotes us to further elucidate the underlying mechanism.

In the present study, we aim to identify the function of lncRNA XIST in promoting GC cell growth and invasion. In addition, we investigate whether XIST affected the biological processes of GC through regulating the expression of miR-497.

## RESULTS

### LncRNA XIST is up-regulated in GC tissues and cell lines and associated with tumor size

**Figure 1:**
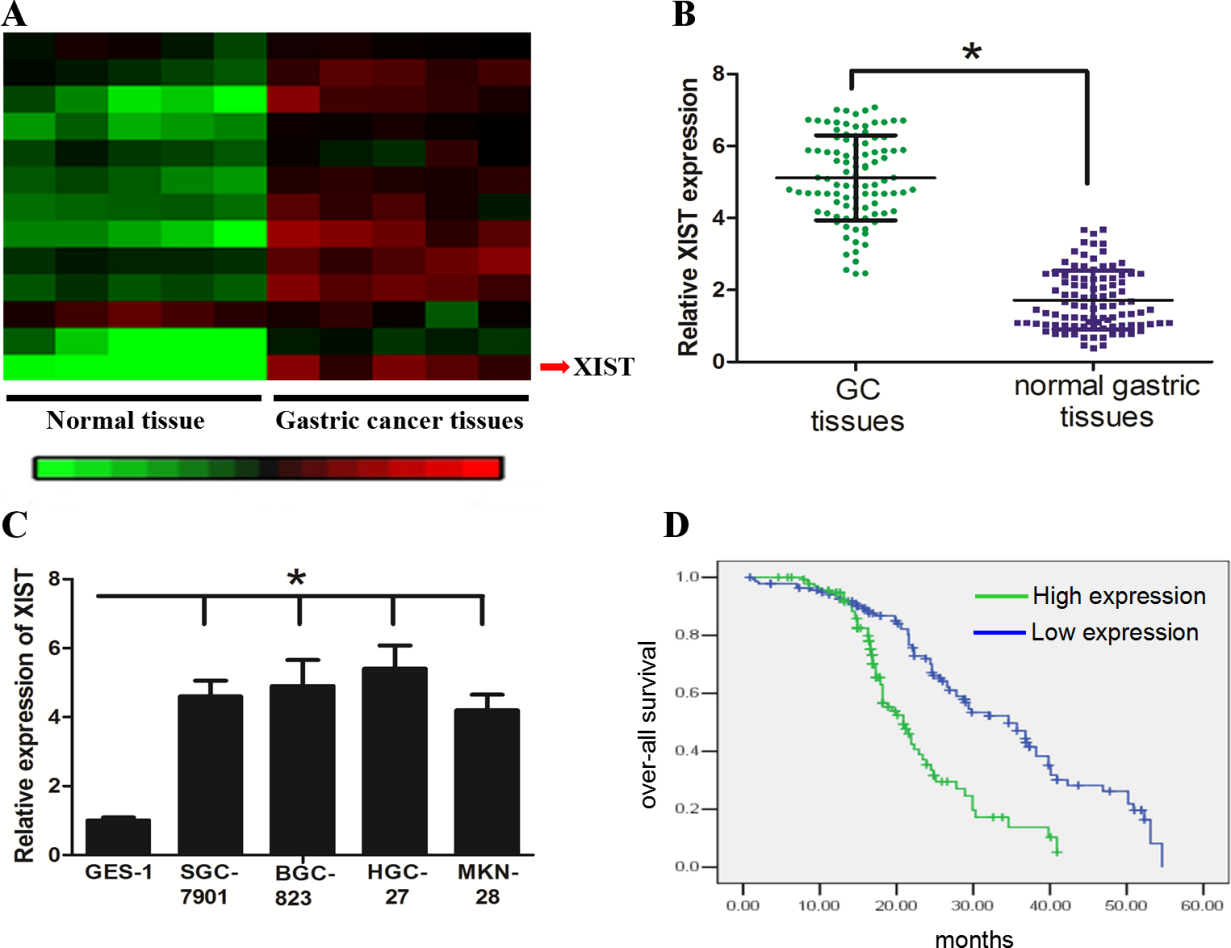
LncRNA XIST expression was up-regulated in GC tissue and associated with short overall survival of GC patients (**A**) and (**B**) XIST expression was significantly increased in the primary GC tissues when compared with the normal counterparts. (**C**) GC cell lines had higher levels of XIST expression than normal gastric cell line GES-1. (**D**) High-level expression of XIST was associated with a shorter overall survival of GC patients (Blue and green curves represent high- and low-expression of XIST, respectively). *represents *P* value < 0.05.

With the Disease-Related Human LncRNA Profiler, we identified that lncRNA XIST was highly expressed in GC tissues when compared with normal gastric tissues (Figure 1A). This result was confirmed in 98 GC and matched-normal tissue samples by qRT-PCR (Figure 1B, *P* < 0.05). We further found that GC cell lines showed higher XIST expression than the normal gastric cell line GES-1 (Figure 1C, *P* < 0.05). Furthermore, we investigated the potential associations between XIST expression and patients’ clinicopathological features (Table 1). Although XIST expression was not associated with parameters such as age (*P* = 0.175), gender (*P* = 0.651), Lauren histotype (*P* = 0.934) and *Helicobacter pylori* infection (*P* = 0.573), high XIST expression was significantly correlated with tumor size (*P* = 0.006), lymph node metastasis (*P* = 0.002) and late clinical stage (*P* = 0.005). In addition, Kaplan-Meier analysis revealed that high-level expression of XIST was associated with a shorter overall survival time in patients with GC (Figure 1D, *P* < 0.05).

**Table 1:** Associations between lncRNA XIST expression and patients’ clinicopathological features

Variable	No. of patients	XIST low expression	XIST high expression	*P* value
**Age**				
< 60	45	21	24	0.175
≧ 60	53	32	21	
**Gender**				
Male	59	33	26	0.651
Female	39	20	19	
**Tumor size**				
< 3 cm	54	36	18	0.006
≧ 3 cm	44	17	27	
**Lymph node involvement**				
Absent(pN0)	43	31	12	0.002
Present(pN+)	55	22	33	
**TNM stage**				
I-II	50	34	16	0.005
III-IV	48	19	29	
**Lauren histotype**				
Intestinal	54	29	25	0.934
Diffuse	44	24	20	
**HP infection**				
Yes	58	30	28	0.573
NO	40	23	17	

### LncRNA XIST inhibition decreased GC cell proliferation and invasion

**Figure 2:**
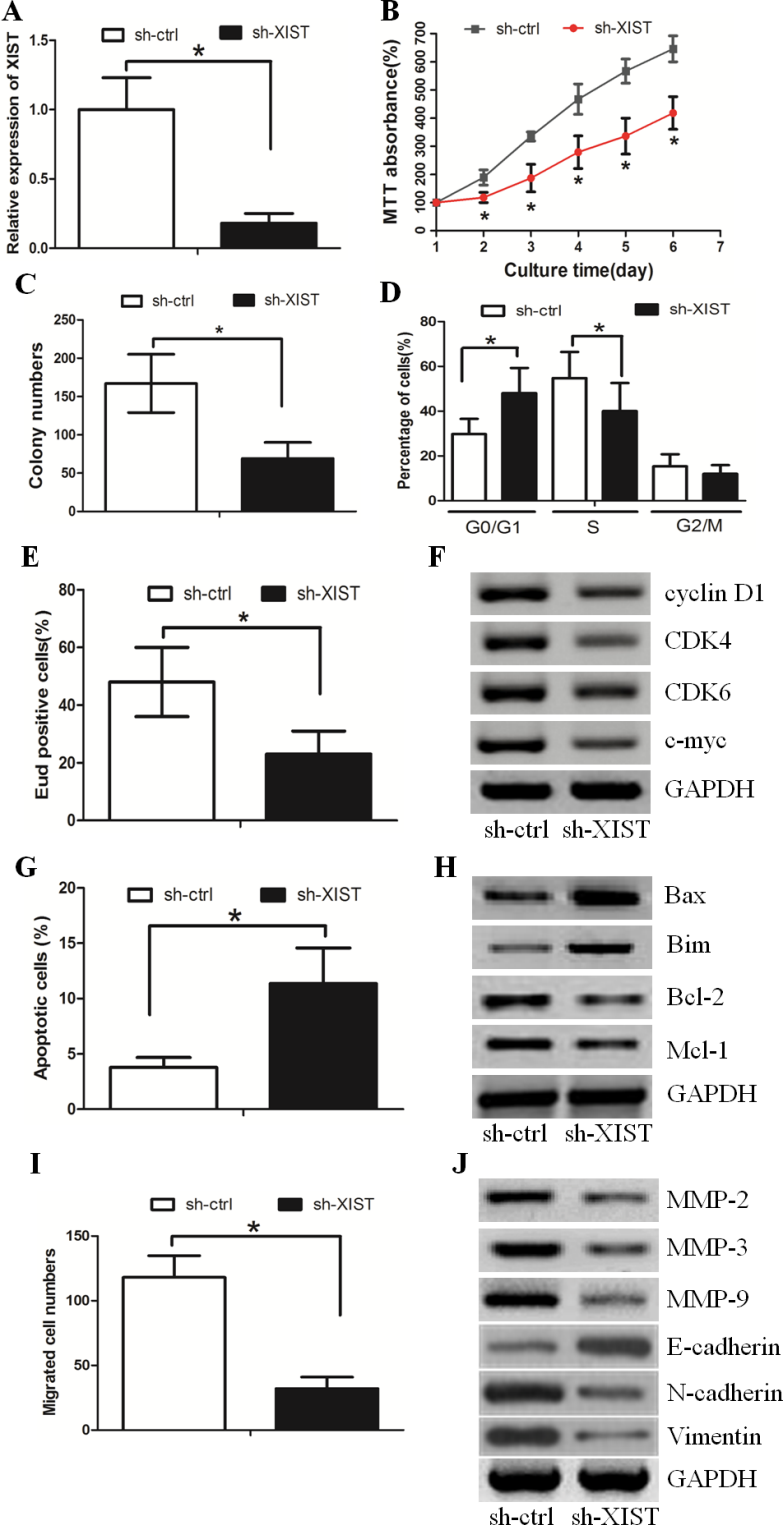
XIST inhibition decreased HGC-27 cell proliferation and invasion (**A**) XIST expression in HGC-27 cells transduced with control shRNA vector (sh-ctrl) or XIST shRNA vector (sh-XIST). (**B**) The MTT assay revealed that XIST down-regulation significantly decreased cell proliferation. (**C**) Colony formation assay demonstrated that oncogenic survival was significantly decreased in sh-XIST cells compared with sh-ctrl cells. (**D**) sh-XIST cells displayed a significantly higher frequency of cells at the G1 phase and a lower frequency of cells at S phase. (**E**) The number of cells incorporating EdU was significantly decreased in the sh-XIST group when compared with the sh-ctrl group. (**F**) XIST down-regulation affected the expression of G1/S phase checkpoint proteins. (**G**) XIST down-regulation increased the proportion of apoptosis in HGC-27 cells. (**H**) The alteration of pro-apoptotic proteins (Bax and Bim) and anti-apoptotic proteins (Bcl-2 and Mcl-1) in sh-XIST cells. (**I**) XIST down-regulation decreased the HGC-27 cell invasion ability, as revealed by the boyden assay. (**J**) The expression levels of MMP-2, MMP-3, MMP-9, N-cadherin and Vimentin protein were lower in sh-XIST cells. The expression level of E-cadherin was higher.

Since XIST expression was the highest in the HGC-27 GC cell line, we established HGC-27 cells in which we stably knocked down XIST (sh-XIST). Cells treated with a negative control were named sh-ctrl (Figure 2A). The MTT assay revealed that knocking down XIST significantly decreased cell proliferation when compared with sh-ctrl cells (Figure 2B). In parallel, the results of the colony formation assay demonstrated that oncogenic survival was significantly decreased in sh-XIST cells when compared with sh-ctrl cells (Figure 2C, Supplementary Figure S1A). Flow cytometry analysis showed that sh-XIST cells displayed a significantly higher frequency of cells at the G1 phase and a lower frequency of cells at the S phase (Figure 2D, Supplementary Figure S1B). This indicated that XIST promoted cell cycle progression from the G1 phase to the S phase. Similarly, the EdU incorporation assay revealed that the number of cells incorporating EdU was significantly decreased in the sh-XIST group when compared with the sh-ctrl group (Figure 2E, Supplementary Figure S1C). In addition, we detected that the expression of G1/S phase checkpoint proteins such as cyclin D1, CDK4, CDK6 and c-myc was significantly decreased in sh-XIST cells (Figure 2F).

FACS analysis revealed that XIST down-regulation could induce cell apoptosis in GC (Figure 2G, Supplementary Figure S1D). We also detected the up-regulation of pro-apoptotic proteins (Bax and Bim) and down-regulation of anti-apoptotic proteins (Bcl-2 and Mcl-1) in sh-XIST cells (Figure 2H).

The boyden assay revealed that XIST down-regulation inhibited GC cell invasion (Figure 2I Supplementary Figure S1E). Consistent with this data, the expression levels of well-defined invasion protein markers (including MMP-2, MMP-3 and MMP-9) markedly decreased in sh-XIST cells (Figure 2J). Furthermore, XIST down-regulation increased the expression level of E-cadherin and decreased the expression levels of N-cadherin and Vimentin (Figure 2J). These data suggested that lncRNA XIST may promote GC cell invasion by inducing the EMT phenotype. Taken together, these data suggested that XIST promoted GC cell proliferation and invasion *in vitro*.

### Reciprocal repression between lncRNA XIST and miR-497 expression in GC

**Figure 3:**
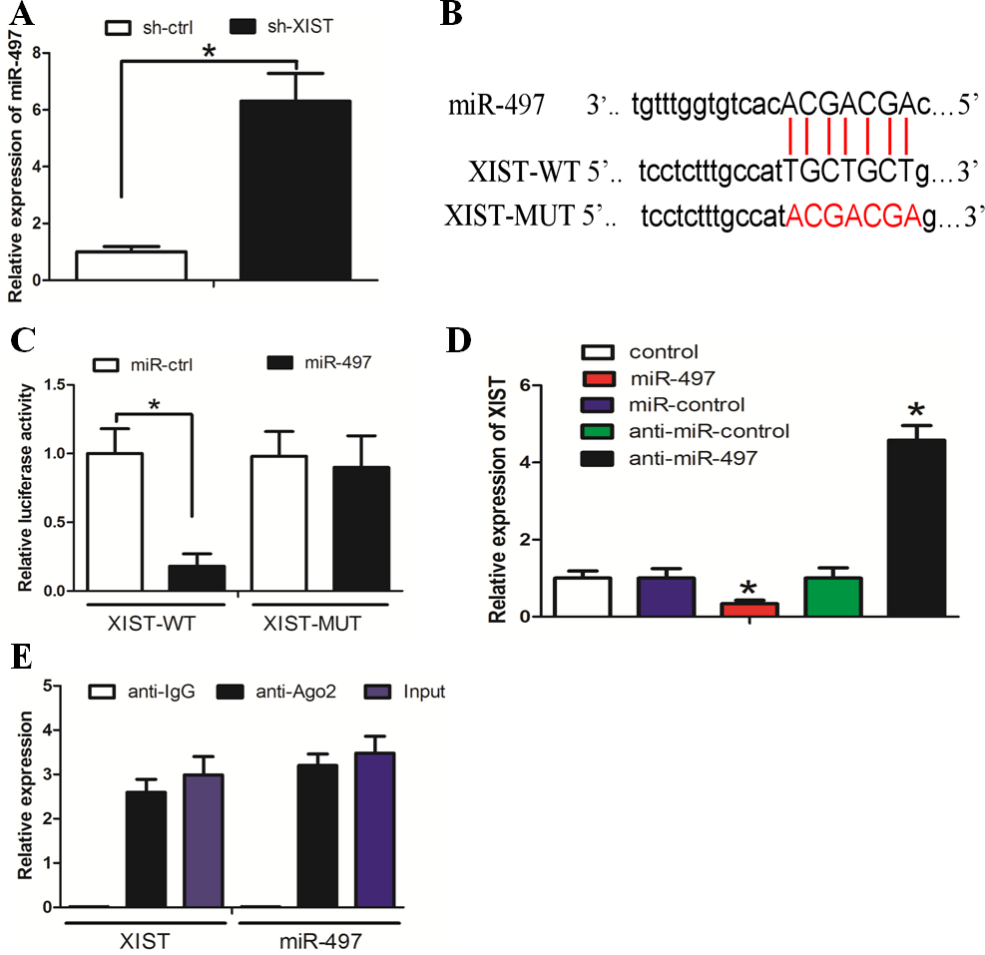
Reciprocal repression between lncRNA XIST and miR-497 in HGC-27 cells (**A**) MiR-497 expression was increased in sh-XIST cells when compared with sh-ctrl cells. (**B**) The binding sites of miR-497 on XIST. (**C**) Co-transfection of miR-497 and XIST-Wt strongly decreased the luciferase activity, while co-transfection of miR-control and XIST-Wt did not change the luciferase activity. Co-transfection of miR-497 and XIST-Mut did not change the luciferase activity either. (**D**) MiR-497 decreased XIST expression. (**E**) XIST and miR-497 were enriched in Ago2 immunoprecipitates relative to control IgG immunoprecipitates.

We searched for miRNAs with complementary base paring with XIST utilizing the online software program starbase v2.0 (http://starbase.sysu.edu.cn/mirLncRNA.php). From the result we focused on miR-497, a known tumor suppressor that suppresses cancer cell proliferation and invasion (Figure 3B). The RT-PCR assay showed that miR-497 expression was increased in the sh-XIST group when compared with the sh-ctrl group (Figure 3A). To further investigate whether XIST was a functional target of miR-497, we cloned the predicted miR-497 binding site of XIST (XIST-Wt) and a mutated binding site (XIST-Mut) into a reporter plasmid. The results showed that co-transfection of miR-497 and XIST-Wt strongly decreased the luciferase activity, while co-transfection of miR-NC and XIST-Wt did not change the luciferase activity. Interestingly, co-transfection of miR-497 and XIST-Mut did not change the luciferase activity either (Figure 3C, *P* < 0.05). As shown in Figure 3D, XIST expression was decreased in cells treated with miR-497, whereas the expression in cells treated with anti-miR-497 was increased (*P* < 0.05). Taken together, these data suggested that miR-497 could directly bind to XIST and decrease XIST expression.

It is documented that miRNAs exert their gene silencing functions through a ribonucleoprotein complex called the RNA induced silencing complex (RISC) [23]. The core component of the RISC is Ago2. A RIP experiment followed by RT-PCR demonstrated that XIST and miR-497 were enriched in Ago2 immunoprecipitates relative to control IgG immunoprecipitates (Figure 3E, *P* < 0.05). These data indicated that both XIST and miR-497 are probably in the same RISC complex, consistent with our bioinformatic analysis and luciferase assay.

### miR-497/MACC1 axis mediated the effect of lncRNA XIST on cell growth in GC

We asked whether the effect of XIST on cell growth and invasion was mediated by miR-497. The MTT and colony formation assays showed that knockdown of XIST significantly inhibited the growth of GC cells, while anti-miR-497 treatment rescued the effect (Supplementary Figure S2A and S2B). In addition, the alterations in cell cycle distribution, apoptosis and invasion, caused by XIST down-regulation, were also rescued by anti-miR-497 treatment (Supplementary Figure S2C, S2D and S2E).

**Figure 4:**
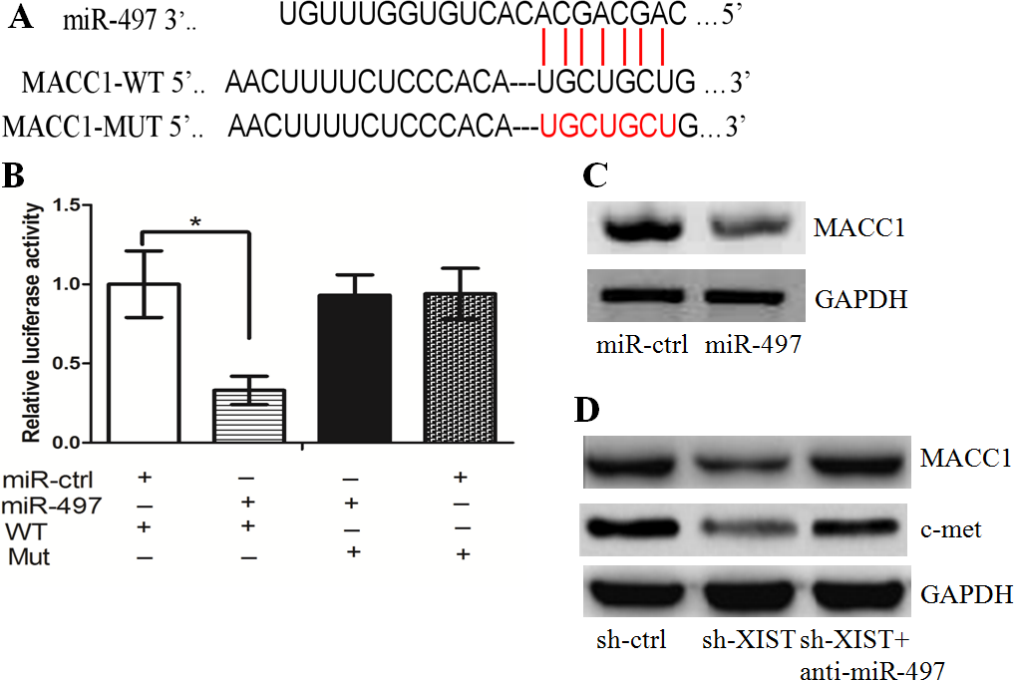
miR-497/MACC1 axis mediated the effect of XIST on HGC-27 cell growth and invasion (**A**) The binding sites of miR-497 on MACC1. (**B**) The luciferase assay showed that cells transfected with miR-497 had less luciferase activity than those transfected with miR-ctrl. (**C**) miR-497 repressed MACC1 protein expression in HGC-27 cells. (**D**) Anti-miR-497 treatment led to the restoration of MACC1 in sh-XIST cells.

MACC1, a key regulator of cell growth and invasion in cancer development, was identified as a down-stream target of miR-497 (Figure 4A). Indeed, the luciferase assay showed that GC cells co-transfected with miR-497 and wt-MACC1-3′-UTR had less luciferase activity than other groups (Figure 4B). The western blot assay demonstrated that miR-497 repressed MACC1 protein expression in GC cells (Figure 4C). In sh-XIST cells, we found that the level of MACC1 was less than that in sh-ctrl cells. However, anti-miR-497 treatment lead to the restoration of MACC1 in sh-XIST cells (Figure 4D). Interestingly, restoration of MACC1 rescued the effect that XIST knockdown had on cell growth, cell cycle distribution, apoptosis and invasion (Supplementary Figure S3A–S3E). We further tested the correlation between the expression levels of lncRNA XIST, miR-497, and MACC1 in clinical samples. It was found that lncRNA XIST expression was negatively correlated with miR-497 expression, while miR-497 expression was negatively correlated with MACC1 expression, respectively (Supplementary Figure S3F). In addition, we tested the potential associations between MACC1 expression and patients’ clinicopathological features (Table 2). It was revealed that high MACC1 expression was significantly correlated with tumor size (*P* = 0.000), lymph node metastasis (*P* = 0.039) and late clinical stage (*P* = 0.000). Taken together, these data suggest that the miR-497/MACC1 axis mediated the effect of XIST on GC cell growth.

**Table 2:** Associations between MACC1 expression and patients’ clinicopathological features

Variable	No. of patients	MACC1 low expression	MACC1 high expression	*P* value
**Age**				
< 60	45	23	22	0.192
≧60	53	34	19	
**Gender**				
Male	59	35	24	0.775
Female	39	22	17	
**Tumor size**				
< 3 cm	54	42	12	0.000
≧3 cm	44	15	29	
**Lymph node involvement**				
Absent(pN0)	43	30	13	0.039
Present(pN+)	55	27	28	
**TNM stage**				
I–II	50	39	11	0.000
III–IV	48	18	30	
**Lauren histotype**				
Intestinal	54	29	25	0.321
Diffuse	44	28	16	
**HP infection**				
Yes	58	38	20	0.076
NO	40	19	21	

### XIST inhibition decreased tumor growth and invasion *in vivo*

**Figure 5:**
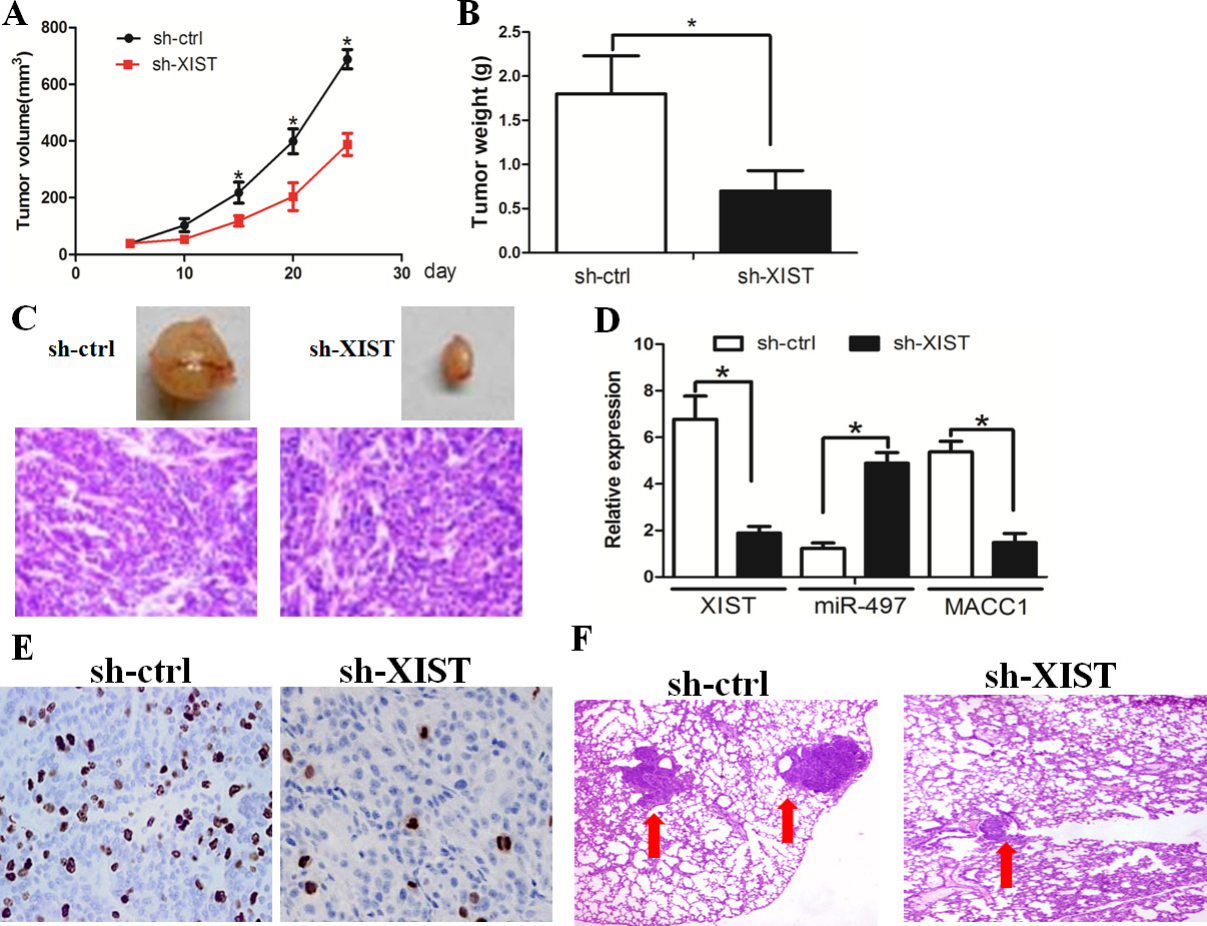
XIST inhibition decreased tumor growth and invasion *in vivo* (**A**) Compared with sh-ctrl cell-derived xenograft tumors, sh-XIST cell-derived xenograft tumors grew more slowly. (**B**) The mean weight of sh-XIST-1 cell-derived xenograft tumors was also significantly less as compared with sh-ctrl cell-derived xenograft tumors. (**C**) The tumors formed in the mice were indicated. (**D**) The expression levels of lncRNA XIST, miR-497, and MACC1 in the samples of xenograft tumor. (**E**) Knockdown of XIST significantly decreased the percentage of Ki-67 positive cells in tumors as compared with the negative control group. (**F**) Knockdown of XIST decreased the metastatic nodules in lung tissue.

We investigated whether knockdown of XIST could inhibit tumor growth *in vivo*. Compared with sh-ctrl cell-derived xenograft tumors, sh-XIST cell-derived xenograft tumors grew more slowly (Figure 5A). The mean weight of sh-XIST cell-derived xenograft tumors was also significantly less when compared with sh-ctrl cell-derived xenograft tumors (Figure 5B, C). LncRNA XIST expression was negatively associated with miR-497 expression, while positively associated with MACC1 expression in the tumor tissues (Figure 5D). Then the tumor sections were stained for Ki-67 expression to quantitatively assess the proliferation index in xenograft tumors (Figure 5E). In addition, the *in vivo* metastasis assay revealed that lncRNA XIST down-regulation resulted in a significant decrease in the size of pulmonary metastatic nodules (Figure 5F). Taken together, these data support the growth-promoting effect of XIST *in vivo*.

## DISCUSSION

LncRNAs may serve as effective therapeutic targets for cancer treatment, including breast cancer, prostate cancer, colon cancer and GC. However, only a few lncRNAs have been functionally characterized. For example, abnormal expression of lncRNA NEAT1 has a close relationship to the development of ovarian cancer occurrence, growth, invasion, and metastasis [10]. Dysfunctional expression of XIST may have a pathological role in cancer. LncRNA XIST can promote cancer cell proliferation and invasion in glioma [9]. In addition, lncRNA XIST can be a predictive biomarker for screening non-small lung cancer [24]. Further investigation revealed that XIST acts as an oncogene in non-small cell lung cancer by epigenetically repressing KLF2 expression [25]. LncRNA XIST can act as a molecular sponge of miR-101 to modulate EZH2 and thereby promotes the progression of gastric cancer [26]. In the present study, we revealed that lncRNA XIST was up-regulated in GC patient tissues and cell lines. Interestingly, high XIST expression was significantly correlated with tumor size, lymph node metastasis and late TNM stage. These results stimulated us to investigate the underlying mechanism of XIST in regulating GC cell growth and invasion. The functional study revealed that knockdown of XIST decreased cell proliferation and invasion both *in vitro* and *in vivo*. Further investigation found that XIST promoted cell cycle progression from the G1 phase to the S phase. In addition, XIST may protect GC cells from apoptosis. LncRNA XIST also promoted GC cells invasion ability through increasing the expression of MMP-2, MMP-3 and MMP-9. These data implicate that XIST functions as an oncogene and drives carcinogenesis by promoting cell proliferation and invasion in GC.

Emerging evidence suggests that lncRNAs act as endogenous miRNA sponges that bind to miRNAs and regulate their function. To find out whether XIST served as a miRNA sponge, we performed bioinformatics analysis and found that XIST contained binding sites for several miRNAs. We focused on miR-497, which has been reported to regulate GC cell proliferation and invasion in our previous study [16]. A Luciferase assay indicated that miR-497 could bind to XIST directly by the putative miRNA response element. Furthermore, overexpression of miR-497 suppressed XIST expression, whereas down-regulation of miR-497 induced a reverse result. Interestingly, a XIST knockdown displayed elevated miR-497 expression. The above data suggest that there might be a reciprocal repression between XIST and miR-497. Finally, we found that XIST and miR-497 were in the same RISC complex, suggesting that there was a physical interaction in GC cells. We further investigated whether miR-497 mediated the tumor-suppressive effect of XIST knockdown in GC. Our present data indicate that while the knockdown of XIST decreases GC cell proliferation and invasion, the inhibition of miR-497 could rescue the effects that the knockdown of XIST exerted.

MACC1 is a regulator of HGF/c-Met signaling, which triggers various malignant behaviors in GC [27]. Its expression correlates positively with GC progression, and it is considered to be an oncogene. Deregulation of MACC1 was reported to promote cell proliferation and invasion in GC cells [28] and MACC1 mediated acetylcholine-induced invasion and migration in human GC cells [29]. MACC1 overexpression also upregulated the mesenchymal-epithelial transition factor and thereby promoted GC cell invasion [30]. In addition, MACC1 supports human GC growth under metabolic stress by enhancing the Warburg effect [31]. In this study, MACC1 was identified as a potential target of miR-497 and miR-497 decreased MACC1 expression in GC cells. In addition, we found that XIST increased MACC1 expression through inhibiting miR-497. Interestingly, restoration of MACC1 rescued the effect of the XIST knockdown on cell growth and invasion. We speculate that miR-497 exerts its function mainly through inhibiting MACC1 expression. LncRNA XIST down-regulation also altered the expression levels of EMT markers ( ie. E-cadherin, N-cadherin and Vimentin). We propose that lncRNA XIST may induce an EMT phenotype through increasing the expression level of MACC1. Taken together, our data demonstrate that XIST may exert its function through the miR-497/MACC1 axis.

In summary, we demonstrated that lncRNA XIST was up-regulated in GC tissues and was associated with worse survival of GC patients. In addition, we uncovered that XIST promoted GC proliferation and invasion through the miR-497/MACC1 axis. Our findings might facilitate the development of lncRNA-directed diagnostics and therapeutics against GC.

## MATERIALS AND METHODS

### Patient samples and cell lines

A total of 98 GC and matched-normal tissue samples were obtained from patients of the Affiliated Cancer Hospital of Guangzhou Medical University. Specimens were obtained during surgery, formalin-fixed and embedded in paraffin by standard methods. All GC patients gave written consent to use their tissue samples for research purposes. This study was approved by the Ethical and Scientific Committees of Guangzhou Medical University. Human GC cell lines (SGC-7901, BGC-823, HGC-27 and MKN-28) and normal gastric epithelial cell line GES-1 were obtained from the Institute of Biochemistry and Cell Biology of the Chinese Academy of Sciences (Shanghai, China). All cell lines were maintained at 37°C in a humidified 5% CO_2_ atmosphere in RPMI-1640 medium supplemented with 10% fetal bovine serum, 100 U/mL penicillin, and 100 mg/mL streptomycin.

### RNA isolation and quantitative real-time PCR

Total RNA was extracted with TRIzol reagent (Invitrogen, Carlsbad, California, USA) according to the manufacturer's instructions. For qPCR, 1 μg of total RNA was reverse transcribed to cDNA using a Reverse Transcription Kit (Takara). Real-time PCR analyses were conducted with Power SYBR Green (Takara). The primers to amplify XIST were 5′-CAGACGTG TGCTCTTC-3′ (forward) and 5′-CGATCTGTAAGTCC ACCA-3′(reverse), for miR-497 were 5′- CTCCACTCCTC CCTTTCCTC-3′(forward) and 5′-GCGGTAAGAAGCA GAGCAG-3′(reverse), for U6 were 5′-CTCGCTTCGGCA GCACA-3′ (forward) and 5′-AACGCTTCACGAATT TGCGT-3′ (reverse) and for GAPDH were 5′-AACGTGT CAGTGGTGGACCTG-3′(forward) and 5′-AGTGGGTG TCGCTGTTGAAGT-3′(reverse). The relative expression of each gene was calculated and normalized using the 2^−ΔΔCt^ method relative to the expression of U6 snRNA or GAPDH.

### Disease-related human LncRNA profiler

The Disease-Related Human LncRNA Profiler was purchased from System Biosciences. The detection of the lncRNAs was performed according to the standard protocol.

### Lentivirus production and transduction

Lentiviral shRNA targeting XIST was designed at http://biosettia.com/support/shrna-designer and cloned into the pLV-H1TetO-GFP-Puro vector according to manufacturer's instructions (Biosettia). The viruses were packaged in 293T cells according to standard protocols and the virus particles were harvested 72 h later. The packaged lentiviruses were named sh-XIST and the empty lentiviral vector sh-ctrl was used as a control. HGC-27 cells were infected with virus particles plus 8 μg/ml Polybrene.

### MTT assay, clonogenic assay, EdU assay and apoptosis assay

The MTT assay to assess cell proliferation was carried out as previously described [21]. Briefly, cells were seeded in 96-well plates. MTT (5 mg/ml) was added to each well, followed by an incubation of 4 hours. After the supernatants were removed, DMSO was added to each well and the absorbance was measured at 490 nm.

For the clone formation assay, cells were seeded in 6-well culture plates. After 14 days the cells were stained with hematoxylin solution and colonies containing ≥ 50 cells were counted.

For the EdU assay, cells were seeded in 24-well plates, followed by incubation under standard conditions in complete media. Subsequently, cells were incubated with 40 μM EdU for 2 h before fixation, permeabilization and EdU staining, which was performed according to the manufacturer's protocol. Then the cell nuclei were stained with DAPI (Sigma). For the apoptosis assay, cells were harvested and resuspended in binding buffer. After double staining with FITC-Annexin V and propidium iodide (PI) using the FITC Annexin V Apoptosis Detection Kit I (Ruibo, Guangzhou, China), cells were analyzed using a FACScan^®^ flow cytometer equipped with Cell Quest software (BD Biosciences, San Jose, CA, USA) according to manufacturer's instructions.

### Oligonucleotide transfection

Synthesized RNA duplexes of miRNA control, miR-497 and anti-miR-497 were obtained from Ribobo (Guangzhou, China). pcDNA-3.1-MACC1 was purchased from Biosettia Inc. (Biosettia, San Diego, USA). Oligonucleotide transfection was performed with Lipofectamine 2000 reagent (Invitrogen) according to manufacturer's instructions.

### Western blot assay

Briefly, equal amounts of protein were resolved by SDS-PAGE and transferred to a polyvinylidene fluoride (PVDF) membrane. The membranes were blocked in 5% non-fat skim milk/TBST, followed by incubation with primary antibodies at 4°C overnight. The primary antibodies MACC1 (Lot NO. ab106579), cyclinD1 (Lot NO. ab16663), CDK4 (Lot NO. ab108357), CDK6 (Lot NO. ab151247), c-myc (Lot NO. ab32072), Bax (Lot NO. ab77566), Bim (Lot NO. ab32158), Bcl-2 (Lot NO. ab32124) and Mcl-1 (Lot NO. ab32087) were purchased from Abcam. The used concentration was 1:500. The primary antibodies MMP-2 (Lot NO. sc-6838), MMP-3 (Lot NO. sc-9941) and MMP-9 (Lot NO. sc-21733) were purchased from Santa Cruz. The used concentration was 1:300. Subsequently, the membranes were incubated with appropriate secondary antibodies. The levels of protein were detected with enhanced chemiluminescence reagents (Pierce, Rockford, IL).

### RNA-binding protein immunoprecipitation (RIP) assay

RNA-binding protein immunoprecipitation (RIP) assay was performed using the EZ-Magna RIP Kit (Millipore, Billerica, MA, USA) according to the manufacturer's instructions. Briefly, cells were lysed with the use of RIP lysis buffer, followed by an incubation step with RIP buffer containing magnetic beads conjugated with human anti-Ago2 antibody (Millipore) or negative control Normal Mouse IgG (Millipore). Proteinase K was used to digest the protein and then immunoprecipitated RNA was isolated. A NanoDrop spectrophotometer was used to measure the RNA concentration. Purified RNA was subjected to q-RT-PCR analysis.

### Luciferase reporter assay

For the luciferase reporter assay, the 3′-UTR of MACC1 was amplified by PCR and cloned downstream of the firefly luciferase gene in the pGL3 vector (Promega). The vector was named wild-type (wt) 3′-UTR. Site-directed mutagenesis of the miR-497 binding site in MACC1 3′-UTR was performed using the Quick change site-directed mutagenesis kit (Stratagene, Cedar Creek, USA) and named mutant (mut) 3′-UTR. Cells were transfected with wt-3′-UTR or mut-3′-UTR and miR-ctrl or miR-497. The luciferase assay was performed by using the dual Luciferase reporter assay system (Promega) 48 h after transfection.

### *In vivo* tumorigenesis

Logarithmically growing cells (1 × 10^6^) (sh-ctrl and sh-XIST) were subcutaneously inoculated into the left-right symmetric flank of 6-week-old male BALB/c-nu/nu mice. At the end of the experiment, the mice were sacrificed and the harvested tumors were weighed and processed for Ki-67 staining. To perform *in vivo* metastasis assays, 2 × 10^6^ cells were injected into the tail vein of nude mice. Four weeks later, the mice were sacrificed and tumor tissues derived from lungs were dissected and examined. All animal studies were permitted by the Animal Care Committee of Guangzhou Medical University.

### Statistical analysis

SPSS 16.0 and Graph Pad Prism 5.0 software were used for statistical analysis. Data are represented as mean ± SD. Fisher's exact test was used to identify differences between categorical variables. One-way ANOVA or two-tailed Student's *t-test* was used for comparisons between groups. Differences were considered statistically significant when *P* < 0.05.

## SUPPLEMENTARY MATERIALS FIGURES


