# Supplementary material for: Long non-coding RNA XIST promotes cell growth and invasion through regulating miR-497/MACC1 axis in gastric cancer
Source: Oncotarget. 2016 Nov 28;8(3):4125–35. doi: 10.18632/oncotarget.13670 (PMC5354817; doi:10.18632/oncotarget.13670)
Supplement: Supplementary file 1 [file oncotarget-08-4125-s001.pdf]

Long non-coding RNA XIST promotes cell growth and invasion through regulating miR-497/MACC1 axis in gastric cancer

Supplementary Materials

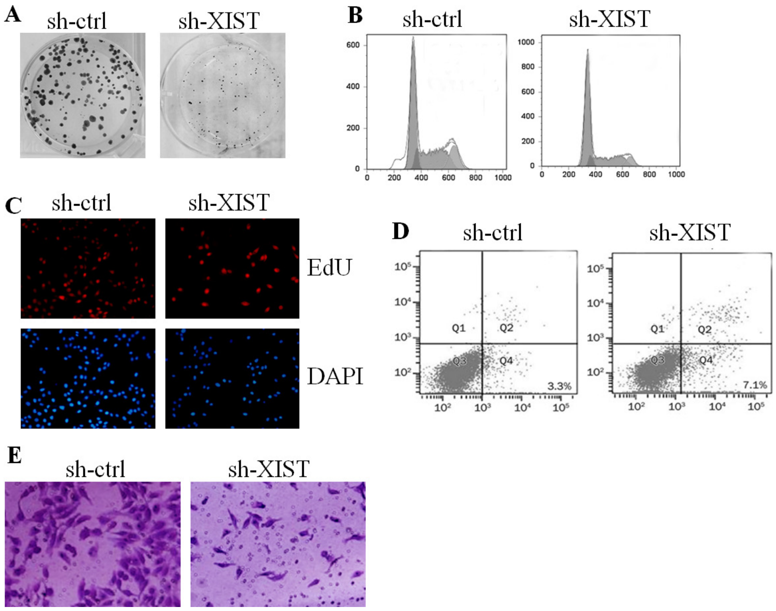

Supplementary Figure S1: XIST down-regulation inhibited HGC-27 cells growth and invasion abilities. (**A**) LncRNA XIST down-regulation decreased colony formation ability of HGC-27 cells. (**B**) LncRNA XIST down-regulation led to cell cycle arrest in the G1 phase. (**C**) The EdU assay revealed that lncRNA XIST down-regulation inhibited HGC-27 cell proliferation. (**D**) Knockdown of XIST led to an increased rate of apoptosis in HGC-27 cells. (**E**) The boyden assay showed that lncRNA XIST down-regulation decreased HGC-27 cell invasion.

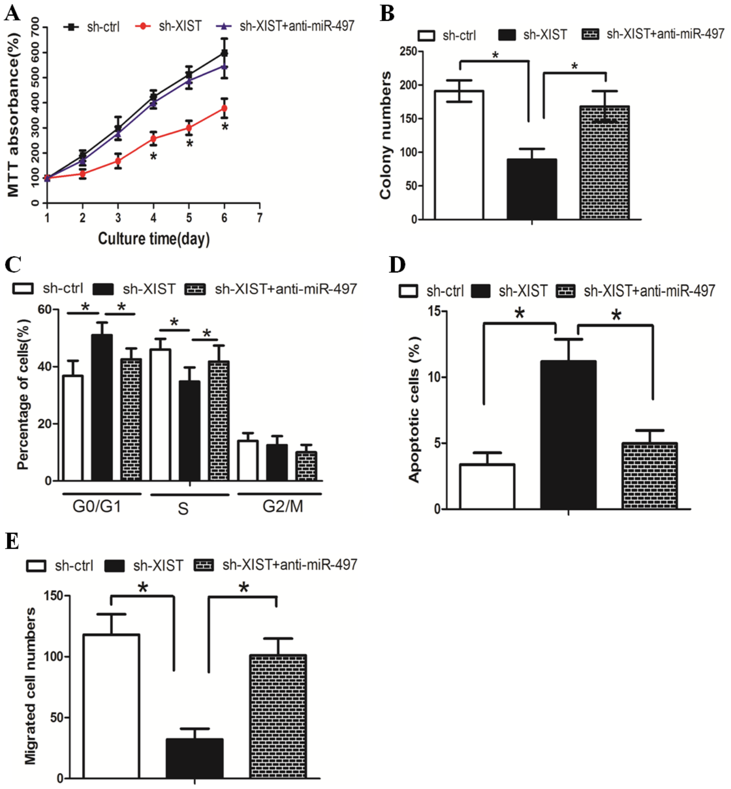

Supplementary Figure S2: Anti-miR-497 treatment rescued the effect of XIST down-regulation on HGC-27 cell growth and invasion. (**A**) and (**B**) MTT and colony formation assays revealed that knockdown of XIST decreased cell proliferation, while anti-miR-497 treatment rescued this effect. (**C**) The cell cycle distribution altered by XIST down-regulation can be counteracted by anti-miR-497 treatment. (**D**) Knockdown of XIST increased apoptosis, while anti-miR-497 treatment counteracted this effect. (**E**) Anti-miR-497 treatment counteracted the effect of XIST down-regulation on HGC-27 cell invasion.

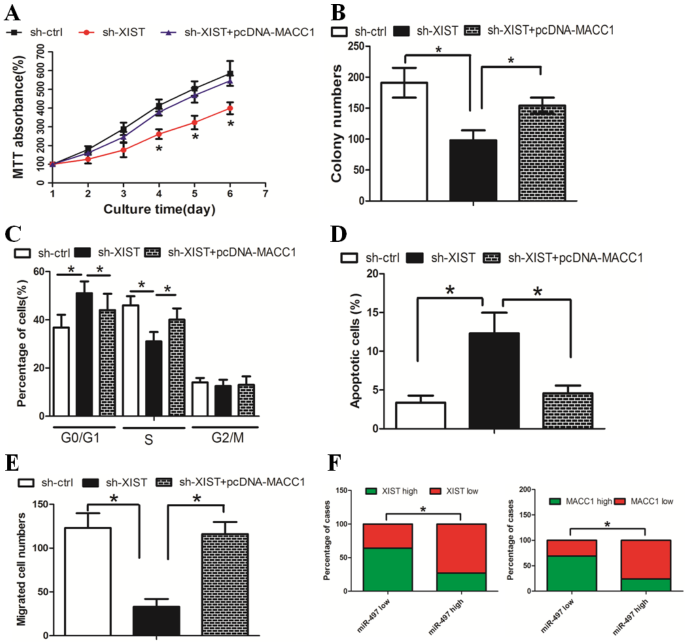

Supplementary Figure S3: Restoration of MACC1 rescued the effect of XIST knockdown on HGC-27 cell growth, cell cycle distribution, apoptosis and invasion. (**A**) and (**B**) MTT and colony formation assays revealed that knockdown of XIST decreased **HGC-27** cell proliferation, while restoration of MACC1 rescued this effect. (**C**) The cell cycle distribution altered by XIST down-regulation can be counteracted by MACC1 restoration. (**D**) Knockdown of XIST increased apoptosis, while overexpression of MACC1 counteracted this effect. (**E**) MACC1 overexpression counteracted the effect of XIST down-regulation on invasion. (**F**) The expression levels of lncRNA XIST, miR-497, and MACC1 in clinical samples.
